# Determinants of Menopausal Symptoms and Attitude Towards Menopause Among Midlife Women: A Cross-Sectional Study in South India

**DOI:** 10.7759/cureus.28718

**Published:** 2022-09-03

**Authors:** Anitha Durairaj, Sriandaal Venkateshvaran

**Affiliations:** 1 Obstetrics and Gynaecology, Velammal Medical College and Hospital, Madurai, IND; 2 Community Medicine, Velammal Medical College and Hospital, Madurai, IND

**Keywords:** attitude towards menopause checklist, menopause rating scale, mid-life women, menopause, post-menopausal symptoms

## Abstract

Introduction

Menopause is the point at which a woman's menstrual periods come to a stop. When a woman goes 12 months without having her period, menopause is diagnosed. Menopause is one stage of midlife that a woman could find simple or challenging to get through. We conducted this study to assess the attitude of women aged over 40 concerning menopause and the determinants of menopausal symptoms.

Methodology

The community-based cross-sectional study was conducted in the villages of Madurai, Tamil Nadu, for six months. We selected four villages and 100 samples using the multistage sampling procedure. Each hamlet had 25 households registered, and we collected the data using the face-to-face interview method. The study included all female participants between the ages of 40 and 60. Those women who had unnatural menopause and women who were on anti-depressant medication and hormone replacement therapy for the past six months were excluded.

Results

The study’s participants had an average age of 52.3 years, and 74% of them had experienced menopause. The menopausal rating scale revealed that around 81.1% of women had somatic symptoms, 70.3% had psychological problems, and 45.9% had urogenital symptoms. People who lived in urban areas, had class 1 socioeconomic status, and had sedentary work showed statistically significant associations with somatic symptoms. Those who lived in urban areas, were professionals by occupation, and did sedentary work showed statistically significant associations with psychological symptoms. The respondents who lived in urban areas had a statistically significant association with urogenital symptoms. We observed a statistically significant correlation between young age and psychological problems. There was a statistically significant correlation between symptoms in all three domains and increased weight.

Conclusion

Middle-aged women have relatively little understanding of menopause. Somatic symptoms are more common in middle-aged women than psychological or urogenital symptoms. Menopausal symptoms are present in almost half of the respondents.

## Introduction

Menopause is the point at which a woman's menstrual periods come to a stop. When a woman goes 12 months without having her period, menopause is diagnosed. Most people have not yet fully understood the reason that menopause is a fact of life. The menopause stage is one midlife stage that a woman could pass through peacefully or with difficulty. There are many beliefs and taboos surrounding this stage of life [1].

Early identification of clinical signs and symptoms can aid in easing women’s discomfort and worries. According to the World Health Organization (WHO), post-menopausal women are those who ceased menstruating a year ago or who do not have periods because of a hysterectomy, an oophorectomy, or both. The WHO also states that the cessation of periods should be about 12 months to confirm menopause.

Women now spend one-third of their lives in this phase because of longer lifespans. There might likely be 130 million old women in India by the end of 2015, demanding a significant quantity of care. Although a few women may handle menopausal symptoms with ease, others could find them to be quite bothersome. The severity of the symptoms will lower a person's overall quality of life for those people. Because of sociocultural considerations, Indian women tend to under-report their symptoms [2,3].

Other studies found that about 20% suffer severe menopausal symptoms, whereas 20% of them gave no symptoms and the remaining have mild symptoms [1]. Significant lifestyle changes among women who exhibit symptoms have resulted in physical and mental illnesses [4].

Presently, no health program in India addresses the unique medical requirements of postmenopausal women. The National Rural Health Mission and Reproductive and Child Health-II program primarily targets women who are still in the reproductive age range, excluding those who have beyond the reproductive stage [5].

In India, we noticed that women’s life expectancies are rising. We conducted this study to assess the attitude of women aged over 40 concerning menopause and the determinants of menopausal symptoms. The current study also estimates the prevalence of menopausal symptoms and assesses their coping strategy.

## Materials and methods

Study design

This was a community-based cross-sectional analytical study. The current study was conducted in the rural field practicing area of Velammal Medical College and Hospital, Madurai. This area comprises about 40,000 population with 12 villages.

Study period and study population

This study had been conducted for six months from 1st June to 31st November 2019. The participants were middle-aged women of age 40 to 60 years living in the field practice area of Velammal Medical College Hospital in Tamilnadu, India.

Ethical clearance

The institutional ethical committee of Velammal Medical College Hospital in Madurai has given its approval for this study (IEC Ref No: VMCIEC 28/2017).

Sampling method and sample size

Multistage sampling was used to select study participants. By employing a simple random sampling technique (using a random number table), four villages were first selected. Then, by using systematic sampling, every tenth house was selected for the research subjects. A nearby household was investigated for the study participant if there were no people in the selected house suiting the eligibility requirements.

The prevalence rate of menopausal symptoms was 89.3%, according to a study done in New Delhi by Singh et al. [6]. Considering this prevalence, we calculated a minimal sample size required for the current study as 100 subjects with a 95% confidence interval and a 6% absolute error. In each village (4 out of 12 villages), about 25 households were registered, and we collected the data using the face-to-face interview method.

Eligibility criteria

Inclusion Criteria

The study included all the women between the ages of 40 and 60 years, who lived in the selected home and expressed their willingness to take part.

Exclusion Criteria

Women who had unnatural menopause, were extremely unwell, were on antidepressants, or had received hormone replacement treatment within the previous six months were all excluded from the study.

Data tool

The data tool had five parts. The first one comprised questions regarding the study participant’s demographic profile. The modified B.G. Prasad scale was used to classify the socioeconomic status of the study participants [7]. The second involved an appraisal of the study participants’ knowledge and awareness of menopause. The third was a standard post-menopausal women’s questionnaire known as the Attitude Towards Menopause (ATM) checklist that was used to gauge middle-aged women’s attitudes toward menopause. The ATM scale assesses women's perceptions of the physical and social changes brought on by aging. Some psychologists believe that menopausal women's psychological issues are a consequence of cultural norms [8]. The fourth part included information about the symptoms of the study participants. Modified Menopausal Rating Scale-11, a standard questionnaire, was used to evaluate these symptoms (MRS). The 11-item measure divides the menopausal symptoms into three categories: physical, psychological, and urogenital symptoms. A Health-Related Quality of Life (QOL) assessment with good psychometric properties is the MRS scale. The use in several countries allowed for the comparison of test characteristics between countries. The reliability measures (consistency and test-retest stability) were determined to be good in all of the countries where data was collected. The validity was assessed in several ways: The internal structure of the MRS scale questions was similar enough across nations to conclude that the scale accurately assessed the same phenomena in women who had complaints [9]. Post-menopausal women’s coping mechanisms were covered in the last section.

Data collection procedure

After obtaining informed consent, the researchers used a face-to-face interview technique to collect the data. The researcher conducted the interview and translated the scale’s questions into the study participant’s everyday language.

Data entry and data analysis

The investigator entered the collected data in Microsoft Excel (Microsoft Corporation, Redmond, WA) and analyzed them using SPSS version 21 (IBM Corporation, Armonk, NY). The mean and standard deviation were used to express all continuous variables. All categorical data were reported as percentages and frequencies. The relationship between menopausal symptoms and socio-demographic factors was determined using the chi-square test.

## Results

About 100 middle-aged women took part in our study. Their average age was 52.3 years. In this survey, 36% of participants were from semi-urban areas, compared to 30% who were from rural areas. Ninety percent of respondents were married while only 21% were employed. The Modified B.G. Prasad scale showed that about 47% of the study’s participants were in socioeconomic class 2. Table 1 displays the study participants’ general characteristics.

**Table 1:** Socio-demographic distribution of the study participants (n = 100)

S. No	Variables		Frequency	Percent
1	Age in years	Mean (Standard deviation)	52.34 (9.415)	
2	Place	Rural	30	30.0
		Semi-urban	36	36.0
		Urban	34	34.0
3	Total number of family members	Less than or equal to five	78	78
		More than five	22	22
4	Education	Secondary education	24	24.0
		Higher secondary education	15	15.0
		Primary education	23	23.0
		Degree holder	14	14.0
		No formal education	24	24.0
5	Socio-economic status (according to the modified B.G. Prasad scale)	Class 1	9	9.0
		Class 2	47	47.0
		Class 3	22	22.0
		Class 4	17	17.0
		Class 5	5	5.0
6	Occupation	Housewife	79	79.0
		Working	21	21.0
7	Marital status	Married	90	90.0
		Unmarried	1	1.0
		Widow	9	9.0
8	Parity	Primi	11	11.0
		Gravida 2	45	45.0
		Gravida 3	35	35.0
		Multi-gravida	9	9.0
9	Level of physical activity	Little	11	11.0
		Moderate	79	79.0
		Strenuous	10	10.0

More than half of the respondents in the survey were aware of what menopause is, but more than half of them were unsure of what to do when they reach it. About 70% of participants were unaware of the long-term menopausal adverse effects. Tables 2, 3 provide information on the study participants’ understanding of menopause.

**Table 2:** Description of qualitative data about menopause among the study participants (n = 100) (multiple options)

S. No	Questions	Answers given by study participants	Frequency	Percent
1	What is menopause according to you?	About age and amenorrhea	5	5.0
		Cessation of menstruation	56	56.0
		End of reproductive capacity	2	2.0
		Hormonal change	7	7.0
		It is normal	4	4.0
		No idea	26	26.0
2	At what age a woman attains menopause?	40 to 45 Years	29	29.0
		45 to 50 Years	56	56.0
		50 to 55 years	5	5.0
		Above 55 Years	5	5.0
		No idea	5	5.0
3	What is the cause of menopause?	Anemia	8	8.0
		Depletion of ovum	1	1.0
		Don't know	38	38.0
		Physiological and hormonal change	17	17.0
		Old age	36	36.0
4	What is the treatment for menopausal symptoms?	Health tonics or drugs	17	17.0
		Consulting doctor	6	6.0
		Don't know	52	52.0
		Good food and adequate rest	3	3.0
		Hysterectomy	10	10.0
		No treatment is needed	10	10.0
		Yoga	1	1.0
5	How do you know about menopause?	Books	3	3.0
		Friends and relatives	81	81.0
		Health care	6	6.0
		No idea	8	8.0
		Radio and television	2	2.0

**Table 3:** Description of qualitative data about the long-term effect of menopause among the study participants (n = 100) (multiple options)

S. No	System	What are the long-term effects of menopause?	Frequency	Percent
1	Musculoskeletal system	Joint pain	8	8.0
		Body pain	6	6.0
		Osteoporosis	1	1.0
2	Oncogenic	Cancer can occur	2	2.0
3	Psychological	Depression	4	4.0
		Irritability	2	2.0
		Mood fluctuation	2	2.0
4	Physical	Weakness	5	5.0
		Weight gain	3	3.0
		Weight loss	1	1.0
5	Other systemic problems	Heart problem	5	5.0
		Vision problems	2	2.0
6	No idea about long-term effects		70	70.0

The ATM checklist was used to evaluate the study subjects' attitudes. Table 4 presents the outcomes.

**Table 4:** Distribution of study participants according to the Attitude Towards Menopause (ATM) checklist (n = 100)

S.No	Questions in the ATM^*^ checklist	Agree	Disagree	Neither
1	After menopause, women feel free to do things for herself	26	43	31
2	Women generally feel better after menopause	30	44	26
3	Women generally become calm and happier after menopause	39	40	21
4	Women have a broader outlook on life after menopause	26	39	35
5	Life is more interesting for women after menopause	38	25	37
6	Women's body only changes after menopause but not herself	41	43	16
7	Women get more confidence in themselves after menopause	44	28	28
8	Going through menopause doesn't change women	45	31	24
9	Difference between menopausal and menstruating women - they get periods	28	51	21
10	Women should see a doctor at menopause	22	48	22
11	Menopause is the biggest change in a woman's life	18	64	18
12	Women are concerned about how their husband feels about them after menopause	36	43	21
13	Menopause is an unpleasant experience	43	39	18
14	Menopause is a disturbing thing that women naturally dread	41	29	30
15	Women should expect some trouble during menopause	31	47	22
16	It is no wonder women feel down the dumps during menopause	37	46	17
17	Changes in the body that women cannot control cause all the trouble at menopause	31	41	28
18	Women worry about losing their minds during menopause	39	38	23
19	Women think of menopause as the beginning of the end	38	37	25
20	Every woman is depressed about menopause	33	43	24
21	Women use menopause changes as an excuse for getting attention	42	21	37
22	After menopause, women don't consider themselves real women	46	32	22

**Figure 1:**
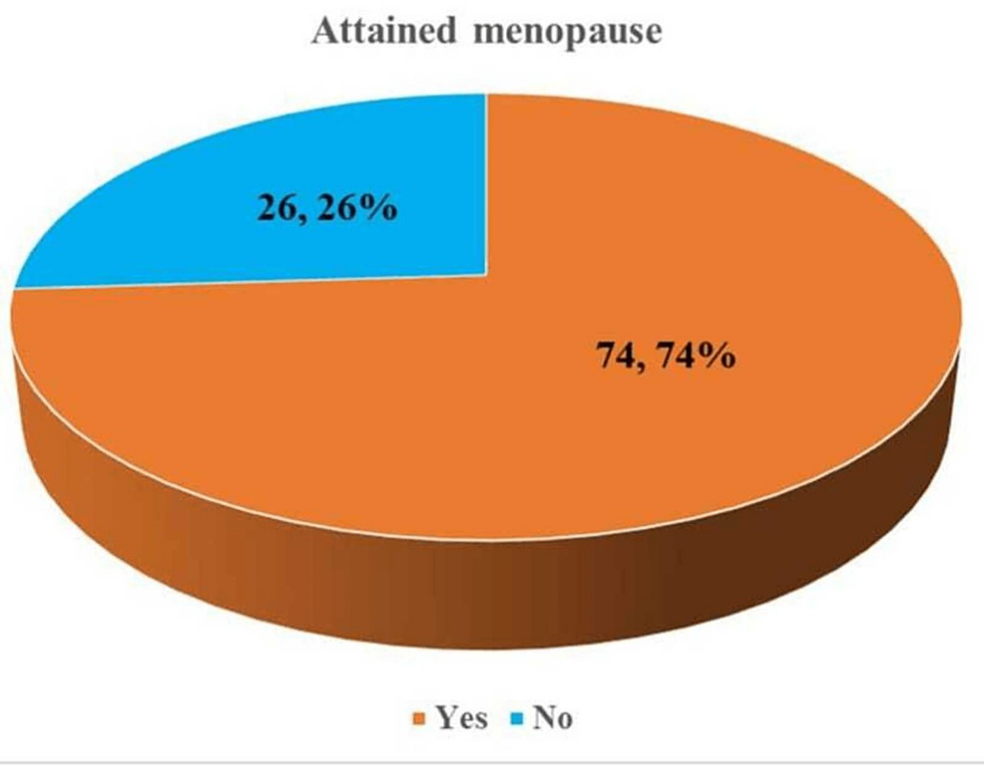
Distribution of the study participants according to their menopausal status (n = 100)

Approximately 74% of participants in the study reached menopause (Figure 1).

On the menopausal rating scale, approximately 81.1% of women experienced somatic symptoms, 70.3% reported psychological problems, and 45.9% reported urogenital symptoms (Table 5).

**Table 5:** Distribution of study participants according to modified menopausal rating scale (MRS) among women who have attained menopause (n = 74)

S.No	Modified - Menopause rating scale							
	Domains		n	%	Individual Questions		n	%
1	Somatic	Yes	60	81.1	Hot flushes and sweating	Yes	47	63.5
						No	27	36.5
					Heart discomfort	Yes	38	51.3
						No	36	48.7
		No	14	18.9	Sleep problem	Yes	49	66.2
						No	25	34.8
					Pain in the joint	Yes	66	89.1
						No	8	10.9
2	Psychological	Yes	52	70.3	Depressive mood	Yes	48	64.8
						No	26	35.2
					Irritability	Yes	46	62.1
						No	28	37.9
		No	22	29.7	Anxiety	Yes	50	67.5
						No	24	32.5
					Physical and mental exhaustion	Yes	51	68.9
						No	23	31.1
3	Urogenital	Yes	34	45.9	Sexual problems	Yes	25	33.7
						No	49	66.3
					Bladder problem	Yes	33	44.5
		No	40	54.1		No	41	55.5
					Dryness of vagina	Yes	40	54
						No	34	46

To cope with menopause, roughly 37.8% of people changed their diets, and about 29.7% preferred to unwind by listening to music or watching television (Table 6).

**Table 6:** Distribution of study participants according to their choice of coping strategy among women who have attained menopause (n = 74) (multiple options)

S. No	Choice of coping strategy adopted by you to overcome menopause	Frequency	Percent
1	Consult a doctor	18	24.3
2	Dietary changes	28	37.8
3	Physiotherapy	3	4
4	Alternative medicine	4	5.4
5	Relaxation - TV/Reading	22	29.7
6	Physical activity	17	22.9
7	Religious activity	11	14.8
8	Talking to friends/relatives	36	48.6

The chi-square test shows a statistically significant association between urban residence, socioeconomic status classes 1 and 4, and sedentary employment in terms of somatic complaints (as determined by MRS scoring) among research participants. The chi-square test shows a statistically significant association between the urban residences, professionals by occupation, sedentary work, and psychological symptoms of the research participants (as determined by MRS score). Under MRS scoring, there is a significant statistical association between study participants who lived in urban areas and urogenital symptoms, as determined by the chi-square test. Table 7 shows the relationship between the study participant’s residence and the domains of the modified MRS assessment.

**Table 7:** Association between study participant’s place of residence and the domains of the modified menopausal rating scale (MRS) assessment (n = 74)

S. No	Variables			Urogenital		Psychological		Somatic domain	
				No	Yes	No	Yes	No	Yes
1	Place	Rural	n	15	9	10	14	8	16
			%	62.5%	37.5%	41.7%	58.3%	33.3%	66.7%
		Semi-urban	n	18	10	10	18	6	22
			%	64.3%	35.7%	35.7%	64.3%	21.4%	78.6%
		Urban	n	7	15	2	20	0	22
			%	31.8%	68.2%	9.1%	90.9%	0.0%	100.0%
		P-value		0.044		0.037		0.014	
2	Education	10th	n	6	7	2	11	1	12
			%	46.2%	53.8%	15.4%	84.6%	7.7%	92.3%
		12th	n	4	5	0	9	0	9
			%	44.4%	55.6%	0.0%	100.0%	0.0%	100.0%
		5th	n	11	7	7	11	6	12
			%	61.1%	38.9%	38.9%	61.1%	33.3%	66.7%
		Degree	n	5	3	1	7	0	8
			%	62.5%	37.5%	12.5%	87.5%	0.0%	100.0%
		Illiterate	n	13	10	12	11	7	16
			%	56.5%	43.5%	52.2%	47.8%	30.4%	69.6%
		Professional	n	1	2	0	3	0	3
			%	33.3%	66.7%	0.0%	100.0%	0.0%	100.0%
		P-value		0.884		0.017		0.092	
3	Socio-economic status	Class 1	n	4	4	1	7	0	8
			%	50.0%	50.0%	12.5%	87.5%	0.0%	100.0%
		Class 2	n	15	16	11	20	4	27
			%	48.4%	51.6%	35.5%	64.5%	12.9%	87.1%
		Class 3	n	12	7	8	11	8	11
			%	63.2%	36.8%	42.1%	57.9%	42.1%	57.9%
		Class 4	n	6	7	2	11	1	12
			%	46.2%	53.8%	15.4%	84.6%	7.7%	92.3%
		Class 5	n	3	0	0	3	1	2
			%	100.0%	0.0%	0.0%	100.0%	33.3%	66.7%
		P-value		0.411		0.300		0.034	
4	Working status	Housewife	n	34	26	18	42	10	50
			%	56.7%	43.3%	30.0%	70.0%	16.7%	83.3%
		Working	n	6	8	4	10	4	10
			%	42.9%	57.1%	28.6%	71.4%	28.6%	71.4%
		P-value		0.351		1.000		0.447	
5	Marital status	Married	n	37	28	19	46	12	53
			%	56.9%	43.1%	29.2%	70.8%	18.5%	81.5%
		Widow	n	3	6	3	6	2	7
			%	33.3%	66.7%	33.3%	66.7%	22.2%	77.8%
		P-value		0.286		1.000		0.676	
6	Level of physical activity	Sedentary	n	7	3	3	7	0	10
			%	70.0%	30.0%	30.0%	70.0%	0.0%	100.0%
		Moderate	n	27	29	14	42	9	47
			%	48.2%	51.8%	25.0%	75.0%	16.1%	83.9%
		Strenuous	n	6	2	5	3	5	3
			%	75.0%	25.0%	62.5%	37.5%	62.5%	37.5%
		P-value		0.238		0.012		0.003	

An independent T-test of the study participants found a statistically significant association between young age and the severity of psychological symptoms. Using an independent T-test, we found that the study participants’ increased weight had a statistically significant association with symptoms across all three categories. Table 8 depicts the association between study participants’ age and weight and the somatic domain using ratings from the modified MRS.

**Table 8:** Association between the age and weight of the study participants and the somatic domain of modified menopausal rating scale (MRS) scores (n = 74)

S. No	Variables	Presence of symptoms	Somatic domain		Psychological		Urogenital	
			Mean	SD	Mean	SD	Mean	SD
1	Age in years	No	56.21	7.402	58.55	8.192	55.95	8.608
		Yes	54.77	8.786	53.56	8.280	53.97	8.401
		P-value	0.570		0.020		0.322	
2	Weight in Kg	No	50.43	7.653	51.95	7.625	55.73	8.357
		Yes	60.22	9.737	61.08	9.838	61.47	11.163
		P-value	0.001		< 0.001		0.014	

## Discussion

The current study showed that the mean age of the study participants was 52.3 years. The mean age of the faculty at a teaching institute in Hyderabad was 48 years according to a cross-sectional survey [10]. The results of another study conducted in Kerala by Borker et al. showed that the study participants’ average age was 48.26 years [1]. These findings reveal that geographic distribution has a marginal impact on menopausal symptoms.

According to the current study, around 47% of the study’s participants belonged to socioeconomic class 2 according to the Modified B.G. Prasad scale. Similar findings were observed by Senthilvel et al. in their 2018 study in Kochi, Kerala. They revealed a significant statistical association between post-menopausal symptoms and classes 2 and 3 socio-economic status [11]. In contrast to the discussion above, a study conducted in Puducherry in 2018 by Krishnamoorthy et al. reports that 42.2% of the study participants belonged to a low socioeconomic category [12]. The variation in sampling size and research method that the researchers used may be the source of the socioeconomic status disparity among the study population.

The results of the current study show that more than half of people were aware of what menopause is, but more than half of them were unsure of what to do when they reach it. This finding was similar to a study by Yanekkerem et al. in Turkey, who concluded that women had very low awareness rates and higher rates of unfavorable views regarding menopause. They suggest that improving the study participants’ attitudes could increase knowledge of menopause [13].

Our study revealed that around 81.1% of participants had somatic symptoms, 70.3% had psychological problems, and 45.9% had urogenital symptoms, as measured by the menopausal rating scale. In a study conducted in Turkey, Yanekkerem et al. identified a similar pattern of symptom presentation in which they found somatic symptoms were more prevalent than psychological and somatic symptoms [13]. Shukla et al. carried out a similar study in Gujarat and came to the same conclusions. They estimated that 91.5% of the study participants had post-menopausal physical problems [14].

The urogenital findings of our study are significantly lower than that of the study done by Borker et al., which was conducted in Kerala and reports that around one-third of the study participants had urogenital symptoms [1]. In contrast to our data, a study conducted in Lucknow in 2017 by Khatoon et al. discovered that over 70% of participants experience mental exhaustion and roughly 53% have heat flushes (physical symptoms) [15]. In a different study conducted in West Bengal by Karmarkar et al., they observed that roughly 88% of the participants had depression [16]. Another study by Mathew et al. in Uttar Pradesh found postmenopausal women had a 100% prevalence of physical or somatic symptoms [17]. Further study in various contexts is required to better understand these variations in the prevalence of menopausal symptoms among midlife women.

Four out of every five study participants experienced at least one post-menopausal symptom in the present study. Singh et al. showed similar results in New Delhi, where the prevalence of post-menopausal symptoms was 89.3% [6]. This shows how important health services are for postmenopausal women.

Data show a statistically significant correlation between the study participants’ somatic complaints and their urban residence, socioeconomic status in classes 1 and 4, and sedentary employment. In contrast to our findings, a Punjabi study by Singla et al. found that rural women had more physical complaints than urban women [8]. Sharma et al. reached a similar finding in their study conducted in Jammu and Kashmir, which found that women in rural areas had higher post-menopausal symptoms than those in urban areas [18].

Our study found a statistically significant association between the study participants’ psychological symptoms and their location in an urban area, their occupation as professionals, and their sedentary employment. In contrast to our findings, a study conducted in Punjab by Singla et al. discovered that urban women were more likely than rural women to experience mental depression [8]. The sub-division of semi-urban in our study may cause the discrepancy in the aforementioned discussion though this merits additional investigation.

In 2018, a study conducted in Puducherry by Krishnamoorthy et al. evaluated the relationship between postmenopausal women’s quality of life and their sociodemographic factors. They concluded that about one-third of the study participants who were living in urban had a poor quality of life [12].

Strength and limitation

The cross-sectional design of this study shows that there is no temporal relationship between the symptoms and menopause. A single investigator collected all the data through face-to-face interviews, thus minimizing the inter-observer bias. The current study used a random sampling technique to conduct a community-based investigation, which favors the generalizability of the findings to the entire population. Despite the use of conventional scales, most of the data were subjective, which could have affected the study’s validity.

## Conclusions

Middle-aged women have relatively less understanding of menopause. The somatic symptoms are more common in middle-aged women than the psychological and urogenital symptoms. Of those studied in the current article, menopausal symptoms are present in around half. Urban dwellers, sedentary workers, young people, and people who have gained weight are at a higher risk of developing menopausal symptoms. Therefore, there is a constant need to increase knowledge of menopause. When screening middle-aged women for menopausal symptoms, gynecologists might take the aforementioned risk factors into account.
